# Preliminary report on bycatch fish species collected from the Tokyo Submarine Canyon, Japan

**DOI:** 10.3897/zookeys.843.32410

**Published:** 2019-05-09

**Authors:** Yusuke Miyazaki, Akinori Teramura, Hiroshi Senou

**Affiliations:** 1 Department of Child Studies and Welfare, Shiraume Gakuen College, 1-830 Ogawa-chou, Kodaira, Tokyo 187-8570, Japan Shiraume Gakuen College Tokyo Japan; 2 Fisheries Laboratory, Graduate School of Agricultural and Life Sciences, The University of Tokyo, 2971-4 Benten-jima, Nishi, Hamamatsu, Shizuoka 431-0214, Japan The University of Tokyo Shizuoka Japan; 3 Kanagawa Prefectural Museum of Natural History, 499 Iryuda, Odawara, Kanagawa 250-0031, Japan Kanagawa Prefectural Museum of Natural History Kanagawa Japan

**Keywords:** deep sea, distributional boundary, Sagami Sea, western Pacific

## Abstract

An ichthyofaunal list of bycatch species was compiled, the fish captured by bottom gill-nets set at approximately 300 m depth in the Uraga Suido Channel central Japan. Fragmentary ichthyofaunal lists are available for this area; these lists have focused on chondrichthyans or commercial actinopterygians, but voucher specimens have not been prepared for museum storage. An initial list of the fish fauna was compiled with vouchers, and seven species not previously recorded from the channel are reported. Most of these species belong to the Class Actinopterygii; *Apristurusplatyrhynchus* (Tanaka, 1909), *Beryxdecadactylus* Cuvier, 1829, *Hoplostethusjaponicus* Hilgendorf, 1879, *Sebastesiracundus* (Jordan & Starks, 1904), *Scalicusamiscus* (Jordan & Starks, 1904), *Atrobuccanibe* (Jordan & Thompson, 1911), and an unidentified species of the eelpout family Zoarcidae. The taxonomic identity of the eelpout and the biogeography of the Uraga Suido Channel are considered. Further research is required to resolve outstanding faunistic issues, but live collections will likely end when the aging fishers who provide the specimens retire. At that point, existing museum collections will become increasingly important for future research. Examination of a collection that may have been previously deposited in the Chiba Prefectural Museum will be essential.

## Introduction

The deep ocean is a frontier for ichthyological exploration. The fish fauna of the deep sea is much less well known than that of shallow coastal zones. Efforts to conserve deep-water fish faunas are essential in the face of threats from anthropogenic disturbance, such as seabed mining (Cuyvers et al. 2018). These efforts are hampered by a dearth of data on deep sea fish diversity.

**Figure 1.**
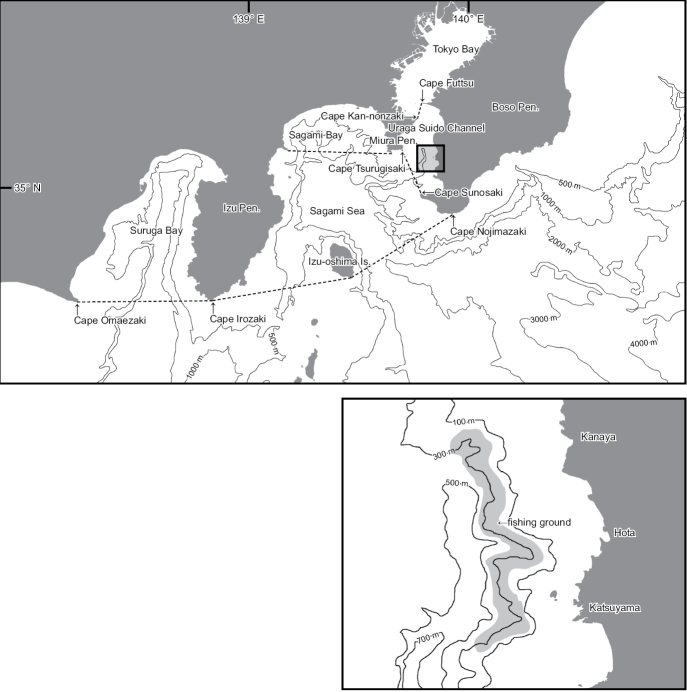
Map showing the study site in the eastern Uraga Suido Channel (extending from Cape Tsurugisaki [south-east of the Miura Peninsula] to Cape Sunosaki [south-west of the Boso Peninsula] and from Cape Kan-nonzaki [east of the Miura Peninsula] to Cape Futtsu [west of the Boso Peninsula]). The channel is included within the broad definition of Tokyo Bay (northward from the Uraga-suido Channel) and Sagami Bay (from Cape Irozaki on the southern Izu Peninsula to Cape Nojimazaki, on the southern Boso Peninsula, including Izu-oshima Island). The channel is not included in the narrow definition of Tokyo Bay.

The Tokyo Submarine Canyon has an unusual hydrography. The shoreward end is located at the mouth of Tokyo Bay (in the narrow sense, defined below). About 31 million people live around the shores of the bay’s basin (Tokyo Bay Environmental Information Center 2017). The maximum water depth in the Uraga Suido Channel at 5–7 km offshore is about 700 m (Fig. 1). The canyon drops off rapidly into the Sagami Trough (> 1000 m deep), and then plunges deeper to the Japan Trench (8020 m maximum depth) (Kato et al. 1985).

Knowledge of the fish fauna in the submarine canyon is fragmentary. Obara et al. (2008) compiled a list of chondrichthyans, which are represented by 41 species belonging to eight orders. Some checklists have included deep-sea species of commercial interest, such as the splendid alfonsino *Beryxsplendens* Lowe, 1834 and the blackthroat seaperch *Doederleiniaberycoides* (Hilgendorf, 1879) (Kohno et al. 2011; Kudo 1997, 2011). The non-commercial ray-finned species occurring in deep water have been little studied, and the few reports available have not provided essential information on voucher specimens, so the records cannot be re-verified.

Data for the ichthyological surveys in the deep waters of the channel have been obtained by examining the bycatch in commercial bottom gill nets. These nets are used to catch the Japanese spider crab, *Macrocheirakaempferi* Temminck, 1836, and the Japanese lobster, *Metanephropsjaponicus* (Tapparone-Canefri, 1873). The nets have been deployed by a single fishing boat, the *Chougorou-maru*, since the 1980s. The first author of this report was able to sail on this boat with a TV crew, and was provided with bycatch specimens as the nets were hauled onboard. The specimens captured included some of the first records from the Uraga Suido Channel. Here, we provide a checklist of the fishes of the Tokyo Submarine Canyon with voucher specimens and photographs. We discuss ichthyofaunal issues in the region.

## Materials and methods

### Definitions of Tokyo Bay and the Sagami Sea

Biogeographical studies of the Uraga Suido Channel have been confused by conflicting definitions of the regions through which it passes, i.e., Tokyo Bay and the Sagami Sea. Some publications have included the channel within Tokyo Bay, while others consider it a component of the Sagami Sea. The broad definition of Tokyo Bay encompasses the Uraga Suido Channel, which includes almost the entire length of the Tokyo Submarine Canyon (Kanou et al. 2010).

Kudo (1997, 2011) and Kohno et al. (2010) used a broad definition of Tokyo Bay, which is divided into the inner and outer sectors. The inner sector lies to the north of a line from Cape Futtsu to Cape Kan-nonzaki. The outer sector lies south of this line and extends to a southernmost boundary line from Cape Sunosaki to Cape Tsurugisaki (Fig. 1). The outer sector of Tokyo Bay in this broad sense corresponds to the Uraga Suido Channel, which is recognized as a major marine traffic lane and a region for the branding of fish products. However, these definitions are not of biological importance (Furota 1997; Senou et al. 2006). The narrow-definition of Tokyo Bay corresponds to the inner sector.

Senou et al. (2006) compiled an ichthyofaunal list of the Sagami Sea, including the Uraga Suido Channel. We follow this work and use the narrow definition of Tokyo Bay.

### Sampling and specimens

The fishing depth was 100–500 m (averaging 200–300 m) below the water surface within the east Tokyo Submarine Canyon, Chiba Prefecture, Japan (Fig. 1; see also Yano et al. 2007; Obara et al. 2008). The fish specimens were provided by two fishers, Mrs Hisao Tejima and Akio Tejima, who operate commercial bottom gill-nets (total 5000 m in length, 1 m in height, and 10 cm mesh size) set on the steep fishing ground (mostly 200–300 m deep). The voyages were undertaken for the production of a TV program series, “Comprehensive Surveys at the Tokyo Bay (original Japanese title: Tokyo-wan Dai-chousa)”, during December 2017, and January and March 2018.

Collected specimens were immediately transferred to a mixture of ambient seawater and ice held in insulated boxes. The fishes were later fixed in 10% formalin, and subsequently preserved in 70% ethanol, except for larger specimens more than approximately 1.0 m total length (TL). Color images were captured after about 1–3 hours fixation. The specimens were deposited in the Kanagawa Prefectural Museum of Natural History, Odawara, Japan (KPM-NI), and in the Museum of Marine Science, Tokyo University of Marine Science and Technology, Tokyo, Japan (MTUF-P). Photographic images of the specimens were deposited in the Image Database of Fishes at the Kanagawa Prefectural Museum of Natural History (KPM-NR).

We were not provided with specimens of fishes with commercial value; these we photographed with an Olympus camera on board the vessel or on the dock. In addition to still shots, we also cut frames from video sequences. These images were also registered to the Image Database of Fishes, and some are available online as “FishPix” (see also Miyazaki et al. 2014).

The systematic arrangement of families, scientific names, and standard Japanese names generally follow Nakabo (2013), with a modification (White et al. 2017).

## Results and discussion

**Figure 2.**
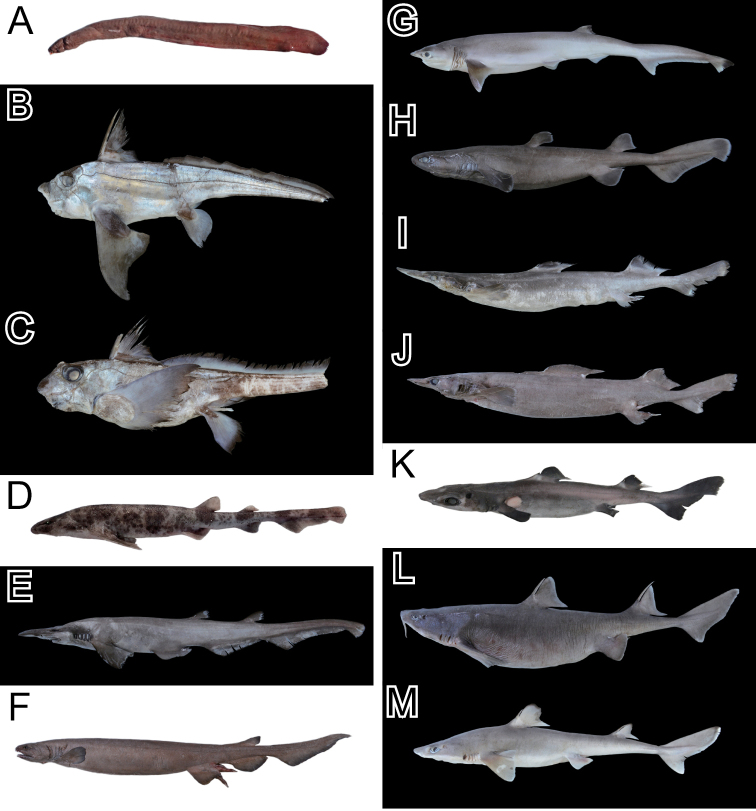
Photos of the voucher specimens of the agnathan and chondrichthyans species collected from the Uraga Suido Channel, the Sagami Sea, Japan. **A**MTUF-P 30681: *Eptatretusatami*, 503 mm TL**B** KPN-NI 47883: *Chimaeraphantasma*, 481 mm TL (tail broken) **C**KPM-NI 46348: *Hydrolagusmitsukurii*, 315 mm TL (tail broken) **D**MTUF-P 30716: *Cephaloscylliumumbratile*, 489 mm TL**E**KPM-NI 46359, *Apristurusplatyrhynchus*, 634 mm TL**F**KPM-NI 46352: *Chlamydoselachusanguineus*, 1258 mm TL**G** KPN-NI 47884: *Heptranchiasperlo*, 910 mm TL**H**KPM-NI 46346: *Dalatiaslicha*, 472 mm TL**I** KPN-NI 47882: *Deaniacalcea*, 656 mm TL**J**KPM-NI 46349: *Deaniahystricosa*, 844 mm TL**K**MTUF-P 30717: *Centrophorusatromarginatus*, 453 mm TL**L**KPM-NI 46356: *Cirrhigaleusbarbifer*, 846 mm TL**M**KPM-NI 46360: *Squalusmitsukurii*, 581 mm TL.

**Figure 3.**
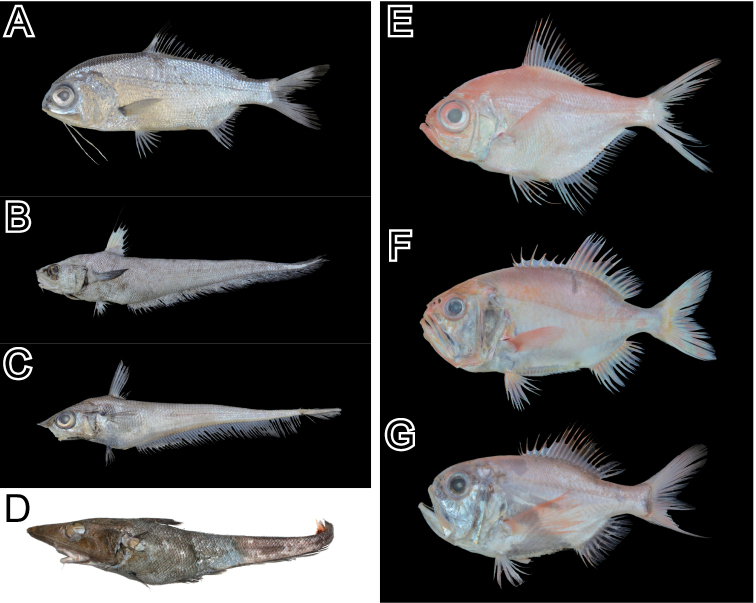
Photos of the voucher specimens of the actinopterygian species (Polymixiiformes, Gadiformes, and Beryciformes) collected from the Uraga Suido Channel, the Sagami Sea, Japan. **A** KPN-NI 47868, *Polymixiajaponica*, 171 mm SL **B**KPM-NI 47880, *Coryphaenoidesmarginatus*, 575 mm TL (tail broken) **C** KPN-NI 47876, *Coelorinchuskishinouyei*, 302 mm TL (tail broken) **D**MTUF-P 30680, *Coelorinchustokiensis*, 772 mm TL (tail broken) **E** KPN-NI 47870, *Beryxdecadactylus*, 210 mm SL **F** KPN-NI 47871, *Gephyroberyxjaponicus*, 204 mm SL **G** KPN-NI 47872, *Hoplostethusjaponicus*, 140 mm SL.

**Figure 4.**
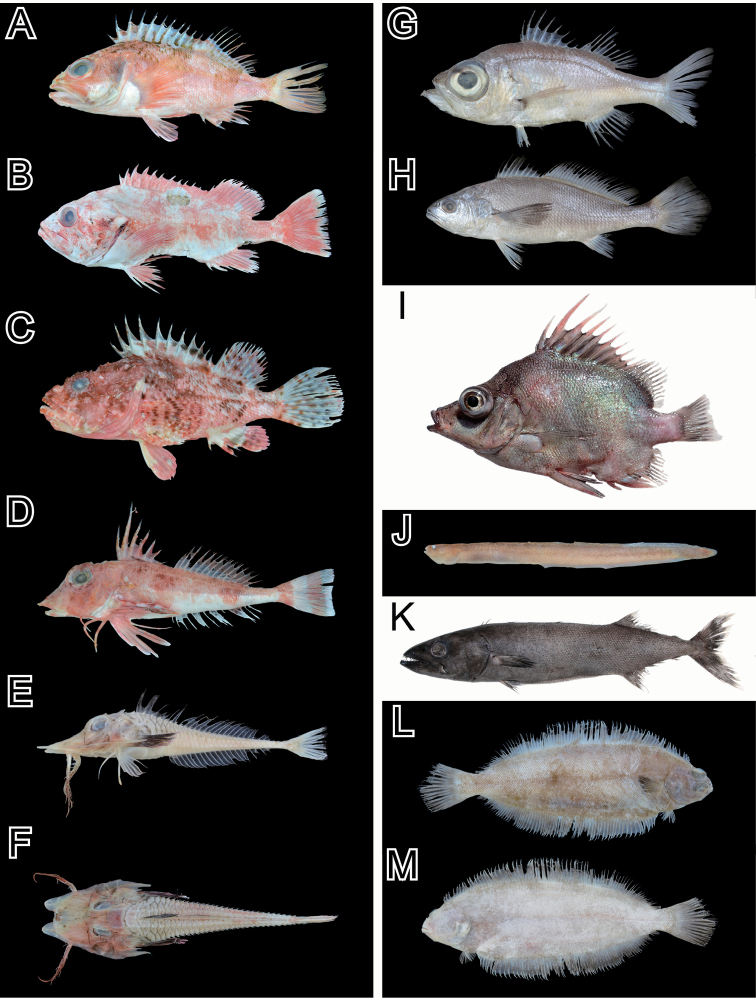
Photos of the voucher specimens of the actinopterygian species (Perciformes, and Pleuronectiformes) collected from the Uraga Suido Channel, the Sagami Sea, Japan. **A** KPN-NI 47875, *Helicolenushilgendorfii*, 146 mm SL **B**KPM-NI 46355, *Sebastesiracundus*, 531 mm SL **C** KPN-NI 47877, *Scorpaenaneglecta*, 158 mm SL **D** KPN-NI 47885, *Lepidotriglaguentheri*, 122 mm SL **E, F** KPN-NI 47887, *Scalicusamiscus*, 179 mm SL **G** KPN-NI 47879, *Malakichthysgriseus*, 150 mm SL **H**KPM-NI 46357, *Atrobuccanibe*, 348 mm SL **I**MTUF-P 30676, *Pentacerosjaponicus*, 162 mm SL **J** KPN-NI 47888, Zoarcidae sp., 78 mm SL **K**MTUF-P 30674, *Ruvettuspretiosus*, 536 mm SL **L, M** KPN-NI 47878, *Tanakiuskitaharae*, 228 mm SL.

**Figure 5.**
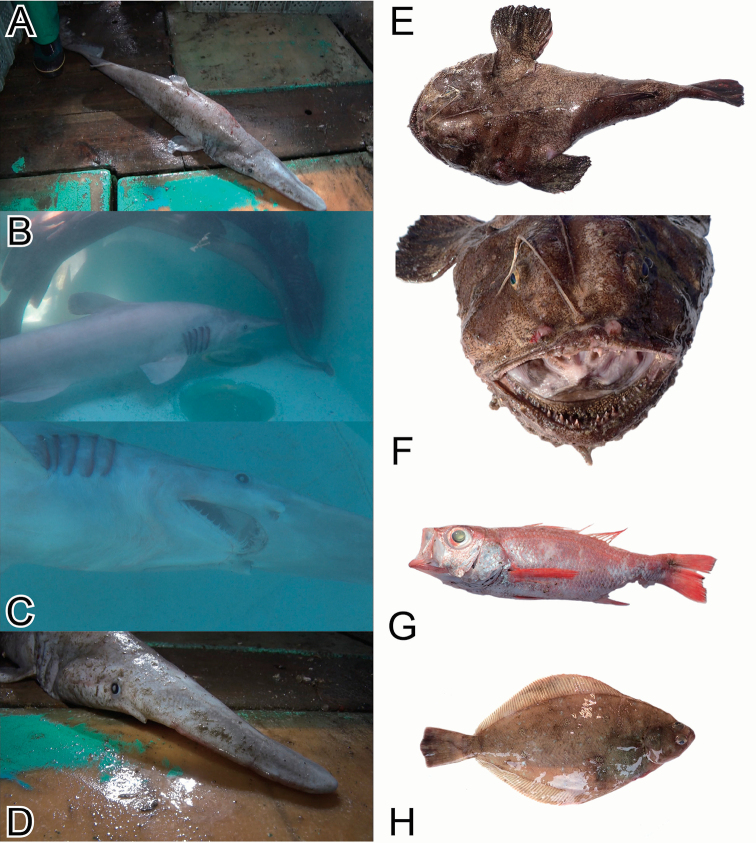
Voucher images deposited to the Image Database of Fishes at the Kanagawa Prefectural Museum of Natural History without specimens collected from the Uraga Suido Channel, the Sagami Sea, Japan. **A–D**KPM-NR 193004A–D, *Mitsukurinaowstoni***E, F**KPM-NR 193003A–B, *Lophiomussetigerus***G**KPM-NR 193002, *Doederleiniaberycoides***H**KPM-NR 193005, *Eopsettagrigorjewi*.

Based on examinations of our voucher specimens (56 individuals) and the photographic images, we identified 36 species in 25 families and 13 orders (Table 1; Figs 2–5). The collection included rare chondrichthyans, such as the frilled shark *Chlamydoselachusanguineus* Garman, 1884 and the goblin shark *Mitsukurinaowstoni* Jordan, 1898, which were previously reported by Yano et al. (2007) and Obara et al. (2008). The four species, *M.owstoni*, *Lophiomussetigerus* (Vahl, 1797), *Doederleiniaberycoides* (Hilgendorf, 1879), and *Eopsettagrigorjewi* (Herzenstein, 1890), were identified from their database photographic images only (Fig. 5); no voucher specimens were deposited in the museums. The compilation included one agnathan, 13 chondrichthyans species (Figs 2, 5), and 21 actinopterygian species (Figs 3–5). Among the actinopterygians, the family Macrouridae was the most speciose (four species). Other families were represented by one or two species.

**Table 1.** An ichthyofaunal list with their vouchers collected by our surveys from the seep-sea area (ranges 100–500 m, average 300 m) of the Uraga Suido Channel, the Sea of Sagami, central Japan. The species with an asterisk indicate the first records with voucher(s) from this area.

Order/Family/Species	Standard Japanese name	Voucher number
Myxiniformes		
Myxinidae		
*Eptatretusatami* (Dean, 1904)	Kuro-nuta-unagi	MTUF-P 30681
Chimaeriformes		
Chimaeridae		
*Chimaeraphantasma* Jordan & Snyder, 1900	Ginzame	KPN-NI 47883
*Hydrolagusmitsukurii* (Jordan & Snyder, 1904)	Aka-ginzame	KPM-NI 46348; MTUF-P 30679
Lamniformes		
Mitsukurinidae		
*Mitsukurinaowstoni* Jordan, 1898	Mitsukurizame	KPM-NR 193004
Carcharhiniformes		
Scyliorhinidae		
*Cephaloscylliumumbratile* Jordan & Fowler, 1903	Nanukazame	MTUF-P 30716
Pentanchidae		
*Apristurusplatyrhynchus* (Tanaka, 1909)*	Herazame	KPM-NI 46359, 46365
Chlamydoselachiformes		
Chlamydoselachidae		
*Chlamydoselachusanguineus* Garman, 1884	Rabuka	KPM-NI 46352, 46354
Hexanchiformes		
Hexanchidae		
*Heptranchiasperlo* (Bonnaterre, 1788)	Edo-aburazame	KPN-NI 47884; MTUF-P 30672
Squaliformes		
Dalatiidae		
*Dalatiaslicha* (Bonnaterre, 1788)	Yoroizame	KPM-NI 46346, 46351, 46353
Centrophoridae		
*Deaniacalcea* (Lowe, 1839)	Hera-tunozame	KPM-NI 47881, 47882
*Deaniahystricosa* (Garman, 1906)	Sagamizame	KPM-NI 46349, 46350, 46364; MTUF-P 30671
*Centrophorusatromarginatus* Garman, 1913	Aizame	MTUF-P 30717
Squalidae		
*Cirrhigaleusbarbifer* Tanaka, 1912	Hige-tsunozame	KPM-NI 46356
*Squalusmitsukurii* Jordan & Snyder, 1903	Futo-tsunozame	KPM-NI 46360–46363, 47886; MTUF-P 30673
Polymixiiformes		
Polymixiidae		
*Polymixiajaponica* Günther, 1877	Ginmedai	KPN-NI 47867–47869
Gadiformes		
Macrouridae		
*Coryphaenoidesmarginatus* Steindachner & Döderlein, 1887	Heri-dara	KPM-NI 46347, 46358, 47880; MTUF-P 30677
*Coelorinchuskishinouyei* Jordan & Snyder, 1900	Mugura-hige	KPN-NI 47876
*Coelorinchusjaponicus* (Temminck & Schlegel, 1846)	Tōjin	MTUF-P 30678
*Coelorinchustokiensis* (Steindachner & Döderlein, 1887)	Miyako-hige	MTUF-P 30680
Lophiiformes		
Lophiidae		
*Lophiomussetigerus* (Vahl, 1797)	Ankō	KPM-NR 193003
Beryciformes		
Berycidae		
*Beryxdecadactylus* Cuvier, 1829*	Nan’yō-kinme	KPN-NI 47870
Trachichthyidae		
*Gephyroberyxjaponicus* (Döderlein, 1883)	Hashikinme	KPN-NI 47871
*Hoplostethusjaponicus* Hilgendorf, 1879*	Hiuchidai	KPN-NI 47872, 47873; MTUF-P 30682
Perciformes		
Sebastidae		
*Helicolenushilgendorfii* (Döderlein, 1884)	Yume-kasago	KPM-NI 47874, 47875
*Sebastesiracundus* (Jordan & Starks, 1904)*	Ōsaga	KPM-NI 46355
Scorpaenidae		
*Scorpaenaneglecta* Temminck & Schlegel, 1843	Izu-kasago	KPN-NI 47877
Triglidae		
*Lepidotriglaguentheri* Hilgendorf, 1879	Kanado	KPN-NI 47885
Peristediidae		
*Scalicusamiscus* (Jordan & Starks, 1904)*	Hige-kihōbō	KPN-NI 47887
Acropomatidae		
*Doederleiniaberycoides* (Hilgendorf, 1879)	Akamutsu	KPM-NR 193002
*Malakichthysgriseus* Döderlein, 1883	Ōme-hata	KPN-NI 47879
Sciaenidae		
*Atrobuccanibe* (Jordan & Thompson, 1911)*	Kuroguchi	KPM-NI 46357
Pentacerotidae		
*Pentacerosjaponicus* Steindachner, 1883	Tsubodai	MTUF-P 30676
Zoarcidae		
Zoarcidae sp.*	Natsushimachojyagenge	KPN-NI 47888
Gempylidae		
*Ruvettuspretiosus* Cocco, 1833	Baramutsu	MTUF-P 30674
Pleuronectiformes		
Pleuronectidae		
*Tanakiuskitaharae* (Jordan & Starks, 1904)	Yanagi-mushigarei	KPN-NI 47878; MTUF-P 30675
*Eopsettagrigorjewi* (Herzenstein, 1890)	Mushi-garei	KPM-NR 193005

We found seven species (Table 1) that had not been included in the previous reports on the fish fauna of the Uraga-suido Channel (Kudo 1997, 2011; Senou et al. 2006; Obara et al. 2008; Kohno et al. 2011): *Apristurusplatyrhynchus* (Tanaka, 1909), *Beryxdecadactylus* Cuvier, 1829, *Hoplostethusjaponicus* Hilgendorf, 1879, *Sebastesiracundus* (Jordan & Starks, 1904), *Scalicusamiscus* (Jordan & Starks, 1904), *Atrobuccanibe* (Jordan & Thompson, 1911), and an unidentified species of the eelpout family Zoarcidae (see below). Miya and Aizawa (1995) briefly reported on the ichthyofauna of the same region based on specimens collected with the fishing gear that we used. This work is reported in an abstract for the “Symposium on Taxonomy, Ecology, and Stocks of Elasmobranchs” held at the Ocean Research Institute, University of Tokyo on November 27–28^th^, 1995. However, neither a species list nor details of voucher specimens were provided, and no publication emerged subsequently. Some of the specimens collected in this 1995 study may have been deposited in the Natural History Museum and Institute, Chiba Prefecture to which they have belonged. Examination of previous collections that may have been deposited in the Chiba Prefectural Museum and other museums will be increasingly important and essential, because live collections will likely end when the aging fishers who provide the specimens retire.

The fishers who provided our specimens are aging, and no successors are likely to take over their operations. Collections will likely cease when the fishing closes down and hence, museum holdings will become increasingly important for faunistic studies.

The specimen that we identified as *Sebastesiracundus* (Fig. 4B) has a black inner surface in the mouth. Nakabo and Kai (2013) indicated that this coloring is characteristic of *Sebastesflammeus* (Jordan & Starks, 1904). However, other morphological traits, such as the patterns of tooth bands, are indicative of *S.iracundus*. A fishing writer also mentioned the same confusion, that is, it is difficult to identify the two species captured from this area based on the external morphology (Shiina 2019). These two species were regarded as conspecifics by Balanov et al. (2004), but genetic differences between the two entities have been reported subsequently (Orr and Hawkins 2008). The Uraga Suido Channel is the southern distribution boundary for these species (Nakabo and Kai 2013), and we provide the southernmost record of *S.iracundus*.

The unidentified eelpout (Fig. 4J) in our collections may be a species shown as “*Andriashevianatsushimae*” by Nishiguchi et al. (2009). However, the name is not available according to Art. 8 of the International Code of Zoological Nomenclature (International Commission on Zoological Nomenclature 1999). There are inconsistencies in the original description (the number of vertebrae in the holotype) and morphological details are inadequate (Hatooka 2013; Shinohara and Takami 2014). Based on further observations of the external and internal morphology of additional specimens collected from Suruga Bay and Sagami Bay, Shinohara and Takami (2014) reported that the undescribed species probably belongs to an undescribed genus. The taxonomic study of this new entity is ongoing.
